# Blood Neutrophil Counts in HIV-Infected Patients with Pulmonary Tuberculosis: Association with Sputum Mycobacterial Load

**DOI:** 10.1371/journal.pone.0067956

**Published:** 2013-07-09

**Authors:** Andrew D. Kerkhoff, Robin Wood, David M. Lowe, Monica Vogt, Stephen D. Lawn

**Affiliations:** 1 School of Medicine and Health Sciences, The George Washington University, Washington, D. C., United States of America; 2 Desmond Tutu HIV Centre, Institute of Infectious Disease and Molecular Medicine, Faculty of Health Sciences, University of Cape Town, Cape Town, South Africa; 3 Clinical Infectious Disease Research Initiative, Institute of Infectious Diseases and Molecular Medicine, University of Cape Town, Cape Town, South Africa; 4 Department of Medicine, Imperial College London, London, United Kingdom; 5 Department of Clinical Research, Faculty of Infectious and Tropical Diseases, London School of Hygiene and Tropical Medicine, London, United Kingdom; Fundació Institut d’Investigació en Ciències de la Salut Germans Trias i Pujol. Universitat Autònoma de Barcelona. CIBERES, Spain

## Abstract

**Background:**

Increasing evidence suggests that neutrophils play a role in the host response to *Mycobacterium tuberculosis*. We determined whether neutrophil counts in peripheral blood are associated with tuberculosis (TB) and with mycobacterial load in sputum in HIV-infected patients.

**Methodology/Principal Findings:**

Adults enrolling in an antiretroviral treatment (ART) clinic in a Cape Town township were screened for TB regardless of symptoms. Paired sputum samples were examined using liquid culture, fluorescence microscopy, and the Xpert MTB/RIF assay. Absolute neutrophil counts (ANC) were measured in blood samples. Of 602 HIV-infected patients screened, 523 produced one or more sputum samples and had complete results available for analysis. Among these 523 patients, the median CD4 count was 169×10^9^/L (IQR, 96–232) and median ANC was 2.6×10^9^/L (IQR, 1.9–3.6). Culture-positive pulmonary tuberculosis was diagnosed in 89 patients. Patients with TB had a median ANC of 3.4×10^9^/L (IQR, 2.4–5.1) compared to 2.5×10^9^/L (IQR, 1.8–3.4) among those who were culture negative (p<0.0001). In multivariable analyses, having pulmonary TB was associated with an adjusted risk ratio (aRR) of 2.6 (95%CI, 1.5–4.5) for having an ANC level that exceeded the median value (ANC ≥2.6×10^9^/L; p = 0.0006) and an aRR of 6.8 (95%CI, 2.3–20.4) for having neutrophilia defined by a neutrophil count exceeding the upper limit of the normal range (ANC >7.5×10^9^/L; p = 0.0005). Patients were then classified into four mutually exclusive groups with increasing sputum mycobacterial load as defined by the results of culture, Xpert MTB/RIF and sputum smear microscopy. Multivariable analyses demonstrated that increasing sputum mycobacterial load was positively associated with blood ANC ≥2.6×10^9^/L and with neutrophilia.

**Conclusions/Significance:**

Increased blood neutrophil counts were independently associated with pulmonary TB and sputum mycobacterial burden in this HIV-infected patient group. This observation supports the growing body of literature regarding the potential role for neutrophils in the host response to TB.

## Introduction

The host response to *Mycobacterium tuberculosis (MTB)* is complex and incompletely understood. While this response is thought to be predominantly mediated by mononuclear leukocytes, evidence has emerged in recent years suggesting that neutrophils may also play a role [1]. It is known that neutrophils are the most commonly infected phagocytic cell in sputum samples in the airways of patients with active tuberculosis (TB) [1], [2]. In a study of direct contacts of patients with proven TB-disease, the risk of TB infection was inversely related to the peripheral blood neutrophil count [3]. Neutrophils appear to not only have a phagocytic role, but they also produce antimicrobial peptides such as cathelicidin LL-37, which has immunomodulatory functions as well as direct activity against MTB [3], [4]. Collectively, these findings suggest that neutrophils may play an important role as part of the innate host response to mycobacteria and contribute to the early control of MTB infection. Conversely, however, in patients with established TB disease, higher peripheral neutrophil counts are associated with delayed mycobacterial clearance from sputum [5] and worse clinical prognosis [6], [7]. Thus, neutrophils may have conflicting roles in the response to MTB that may be related to both mycobacterium-specific and host-specific factors as well as the stage of clinical disease.

In previous studies of new diagnostic assays for HIV-associated TB, we have noticed that there may be an association between HIV-associated TB and increased blood neutrophil counts [8]–[11]. We therefore undertook this careful analysis to determine whether such an association exists. Patients were also categorized into four mutually exclusive groups, reflecting differing sputum mycobacterial burden, to further explore a possible association with sputum bacillary burden using multivariable analyses to control for potentially confounding variables.

## Methods

### Patient Characteristics

The community-based antiretroviral therapy (ART) service in Gugulethu township in Cape Town, South Africa and the burden of TB among its patients have been previously described in detail [12]–[15]. Eligible HIV-infected patients were consecutively recruited from patients newly referred to the clinic for ART initiation. Eligible patients were aged ≥18 years, ART-naïve and did not have a current TB diagnosis. All patients received trimethoprim-sulphamethoxazole prophylaxis. All patients provided written informed consent and the study was approved by the research ethics committees of the University of Cape Town and the London School of Hygiene & Tropical Medicine, UK.

Upon their first visit to the clinic, patients had demographic details recorded, a standardized symptom-screening questionnaire [16] was completed and clinical samples were obtained. Two sputum samples were obtained whenever possible, with at least one sample being induced using nebulised hypertonic saline [17]. Urine samples were collected in sterile containers and were stored at −20°C within 3 hours of specimen collection. EDTA-anticoagulated venous blood samples were obtained. Flow cytometry (Becton Dickinson, Franklin lakes, New Jersey, USA) was used to measure blood CD4 cell counts and plasma viral load was measured using branched DNA technology (Bayer Diagnostics, Tarrytown, New York, USA). Blood absolute neutrophil counts were determined using ADVIA 2120 hematology analyzer (Siemens Healthcare Diagnostics, Erlangen, Germany). The National Health Laboratory Service (NHLS) of South Africa performed all blood tests.

### Laboratory Procedures

Sputum samples were processed in an accredited laboratory. N-acetyl-L-cysteine and sodium hydroxide were used to decontaminate samples, which were then concentrated by centrifugation. Prepared smears were stained with auramine O fluorescent stain for fluorescence microscopy. ‘Smear-positive’ was defined as any smear graded as scanty, 1+, 2+ or 3+. The remaining sputum pellet was then tested by Xpert MTB/RIF assay and liquid culture using Mycobacterial Growth Indicator Tubes (MGIT, Becton Dickinson, Sparks, Maryland, USA). MTBDRplus assay (Hain Lifesciences, Nehren, Germany) was used to identify culture isolates as MTB. Frozen urine samples were defrosted and analysed for the presence of lipoarabinomannan (LAM) using the Clearview TB ELISA (Alere, Waltham, MA, USA) according to manufacturer’s instructions.

The concentrations of gram-negative bacterial endotoxin were measured in blood samples using Limulus Amebocyte Lysate (LAL) QCL-1000 assay (Lonza,Walkersville, MD, USA) via the microplate method. Blood samples were first defrosted to ambient temperature. The microplate was pre-equilibrated to 37°C and 50 µl of serum was added to each microplate well. At the start of the assay, 50 µl of LAL was added to each microplate well and the samples were briefly mixed and incubated for 10 minutes. Next, 100 µl of substrate solution was added to each of wells and they were again briefly mixed and incubated for an additional 6 minutes. After 16 total minutes 100 µl of the stop reagent was added to the wells and the absorbance of each well was read at 410 nm.

### Definitions and Analysis

A confirmed TB case was defined by MTB cultured from one or more sputum sample. In addition, TB cases were categorised according to sputum mycobacterial burden as defined by the results of TB assays on sputum samples: *Xpert-negative/smear-negative* (low mycobacterial burden), *Xpert-positive/smear-negative* (intermediate mycobacterial burden) and *Xpert-positive/smear-positive* (high mycobacterial burden). The normal ANC range was 2.0–7.5×10^9^/L and was based upon NHLS reference ranges. Neutrophilia was defined as an ANC >7.5×10^9^/L, the upper limit of the normal reference range.

We compared medians between two groups using Wilcoxon rank-sum tests; the medians among three or more groups were compared using Kruskal-Wallis tests. Proportions were compared using either chi-squared tests or Fisher’s exact tests as indicated by sample size. Logistic regression analyses were used to identify factors independently associated with culture-confirmed TB and different levels of sputum mycobacterial load. A priori it was determined that continuous variables would be re-coded into categorical variables, either according to approximate median values or clinically logical cut-off points. All variables in the univariable model meeting a cut-off of p≤0.1 were included in the multivariable model. Statistical tests were 2-sided at α = 0.05.

## Results

### Tuberculosis Diagnoses

**Figure 1:**
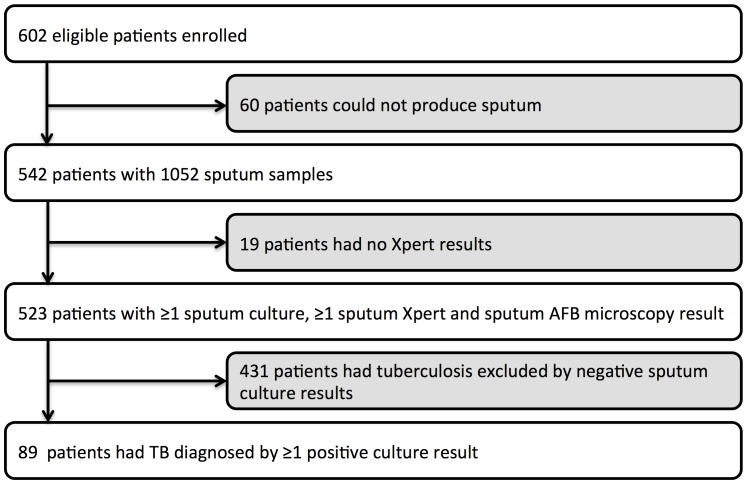
Flow diagram showing patients included in the analysis.

Of 602 patients recruited, 542 (90.0%) produced at least one sputum sample and complete culture and Xpert MTB/RIF results were available for 523 (86.9%) (**Figure 1**). The median CD4 count among those in the cohort (n = 523) was 169 cells/uL (IQR, 96–232). A total of 89 culture-positive pulmonary TB cases were diagnosed (17.0% prevalence [95% CI, 13.9–20.5]) and 431 patients had negative sputum cultures.

Confirmed TB cases were predominantly female, had a median age of 33.4 years (IQR, 28.3–40.4), and a median body mass index (BMI) of 21.2 kg/m^2^ (IQR, 19.2–25.0) (**Table 1**). Additionally, TB cases had a median CD4 cell count of 131 cells/uL (IQR, 55–204), a median plasma viral load of 4.8 log_10_ copies/mL (IQR, 4.4–5.3) and 72 (82.0%) had a positive WHO symptom screen. The median time to culture-positivity was 16 days (IQR, 11–21).

**Table 1:** Characteristics of patients with and without pulmonary TB, and patients with pulmonary TB grouped according to sputum mycobacterial burden.

	Culture negative: no sputum mycobacterial burden (n = 434)	Culture positive (n = 89)	P-value	Low sputum mycobacterial burden (n = 25)	Medium sputum mycobacterial burden (n = 40)	High sputum mycobacterial burden (n = 24)	P-value (comparing 4 sputum mycobacterial load groups)
**Age, median (IQR) (years)**	33.6 (27.9–40.8)	33.4 (28.3–40.4)	0.097	32.1 (28.7–38.0)	35.1 (28.5–41.6)	30.3 (26.4–38.0)	0.6049
**Female, no. (%)**	280 (64.5)	55 (61.8)	0.626	16 (64.0)	22 (55.0)	17 (70.8)	0.584
**Pregnant, no. (%)**	20 (4.6)	0	0.033	0	0	0	0.475
**BMI, median (IQR) (kg/m2)**	23.8 (21.1–27.2)	21.2 (19.2–25.0)	<0.0001	22.1 (20.6–28.5)	21.0 (18.7–23.3)	21.0 (19.0–26.4)	0.0001
**Blood tests** a							
Hemoglobin, median (IQR) (g/dL)	12.2 (10.8–13.5)	10.9 (8.8–12.3)	<0.0001	11.6 (10.4–13.1)	10.8 (8.9–11.9)	9.4 (8.5–11.0)	0.0001
White blood cell count, median (IQR) (cells/uL)	4.8 (3.8–5.9)	5.7 (4.6–7.8)	<0.0001	5.5 (4.1–6.5)	5.5 (4.3–7.8)	5.9 (5.3–10.0)	0.0001
Absolute neutrophil count, median (IQR) (×10^9^/L)	2.5 (1.8–3.4)	3.4 (2.4–5.1)	<0.0001	2.9 (2.4–6.2)	3.3 (2.4–6.2)	4.4 (3.3–7.9)	0.0001
Neutrophilia (ANC >7.5×10^9^/L), no. (%)	7 (1.7)	13 (15.3)	<0.001	1 (4.2)	5 (13.5)	7 (29.2)	<0.001
Absolute lymphocyte count median (IQR) (cells/uL)	1.6 (1.2–2.0)	1.5 (0.9–2.0)	0.1422	1.8 (1.4–2.4)	1.5 (0.7–2.0)	1.3 (0.8–1.8)	0.0285
Endotoxin median (IQR) EU/mL	0.74 (0.59–0.93)	0.72 (0.62–0.93)	0.8239	0.68 (0.52–0.80)	0.87 (0.67–1.11)	0.69 (0.60–0.88)	0.0196
ALT (IU/L)	19 (14–30)	23 (14–34)	0.1758	20 (14–31)	24 (13–34)	23 (14–57)	0.4484
Platelets (platelets/uL)	263 (210–330)	290 (219–384)	0.0404	273.5 (240.5–314.5)	274 (203–393)	363.5 (236–463.5)	0.0663
**CD4 cell counts (cells/uL)** b							
Median (IQR)	173 (111–238)	130.5 (54.5–203.5)	0.0005	189 (137–215)	102 (37–179)	109 (42.5–195)	0.0001
CD4<50	46 (10.6)	20 (22.7)	0.011	3 (12.0)	11 (28.2)	6 (25.0)	0.009
CD4 50–99	53 (12.2)	14 (15.9)		2 (8.0)	8 (20.5)	4 (16.7)	
CD4 100–149	79 (18.2)	17 (19.3)		2 (8.0)	9 (23.1)	6 (25.0)	
CD4 150–199	89 (20.6)	13 (14.8)		7 (28.0)	4 (10.3)	2 (8.3)	
CD4≥200	166 (38.3)	24 (27.3)		11 (44.0)	7 (18.0)	6 (25.0)	
**Baseline viral load, median (IQR) (log copies/mL)** b	4.5 (4.0–5.0)	4.8 (4.4–5.3)	<0.0001	4.4 (4.2–4.7)	5.1 (4.7–5.5)	5.0 (4.7–5.4)	0.0001
**WHO stage at enrolment, no. (%)**							
1 or 2	297 (68.9)	47 (52.8)	0.003	13 (52.0)	22 (55.0)	12 (50.0)	0.033
3 or 4	134 (31.1)	42 (47.2)		12 (48.0)	18 (45.0)	12 (50.0)	
**Positive WHO symptom screen, no. (%)**	289 (66.6)	72 (82.0)	0.004	18 (72.0)	33 (82.5)	22 (91.7)	0.011
**Current cough** ≥**2 weeks, no. (%)**	85 (19.6)	22 (24.7)	0.274	2 (8.0)	9 (22.5)	11 (45.8)	0.011
**Radiological abnormality consistent with TB, no. (%)** c	176 (45.0)	63 (73.3)	<0.001	17 (70.8)	27 (67.5)	19 (86.4)	<0.001
**LAM ELISA positive, no. (%)** d	8 (1.9)	23 (27.4)	<0.001	2 (8.0)	10 (28.6)	11 (45.8)	<0.001
**Time to culture positivity, median (IQR) (days)**	–	16 (11–21)	–	21 (17–25)	16 (13–20.5)	9.5 (8–12)	0.0001

a n = 488,

b n = 520,

c n = 517,

d n = 477.

Compared to patients with negative sputum cultures, TB cases had lower body mass index (BMI), hemoglobin concentration, platelet count and CD4 cell count (**Table 1**). TB cases also had higher viral loads and were more likely to have a positive WHO symptom screen and pulmonary radiographic abnormalities. Blood endotoxin levels were similar in those with and without pulmonary TB.

### Neutrophil Counts in Patients with and without Pulmonary TB

**Figure 2:**
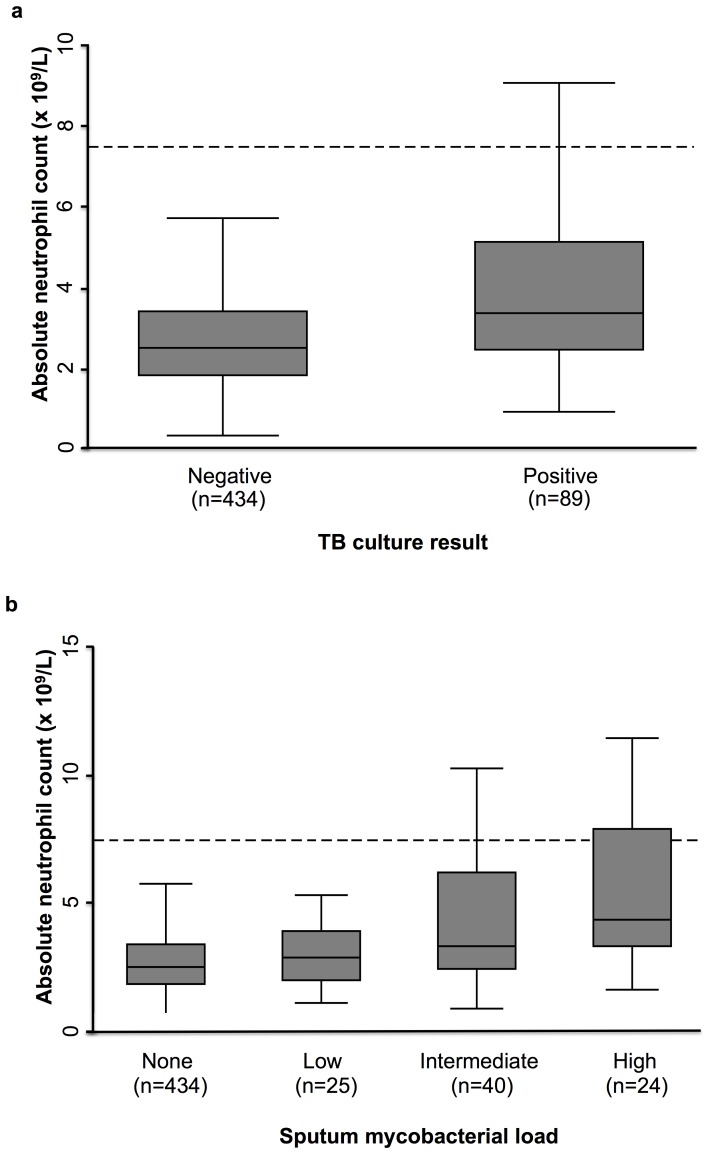
Box and whisker diagram of absolute neutrophil count among those (a) with and without pulmonary TB and (b) and among those with increasing levels of sputum mycobacterial load. The middle of the box represents the median value and the outer box edges represent the 25^th^ and 75^th^ percentiles (IQR). The ends of the whiskers represent the lower and upper adjacent values. Outliers as defined by any data point above the upper adjacent value, were excluded from both plots. The dashed line at ANC = 7.5×10^9^/L represents the upper limit of the normal ANC range, above which is considered neutrophilia.

The median ANC among the total cohort (n = 523) was 2.6×10^9^/L (IQR, 1.9–3.6). However, those with TB had higher blood ANC than those without TB (**Figure 2a**), with median counts of 3.4×10^9^/L (IQR, 2.4–5.1) compared to 2.5×10^9^/L (IQR, 1.8–3.4) (p<0.001). Furthermore, 15.3% (95%CI 8.4–24.7) of patients with pulmonary TB had neutrophilia compared to 1.7% (95%CI 0.7–3.5) among those without pulmonary TB (p<0.001).

In multivariable analyses (**Table 2**), strong associations were observed between culture-confirmed TB and lower blood hemoglobin concentration. Culture-confirmed TB was strongly associated with an ANC greater than the median value and with neutrophilia. Among patients with pulmonary TB, the adjusted risk ratio (aRR) for an ANC above median value was 2.6 (95%CI, 1.5–4.5) compared to those without pulmonary TB (p = 0.0006). Furthermore, among patients with pulmonary TB, the aRR for neutrophilia was 6.8 (95%CI, 2.3–20.4) compared to those without pulmonary TB (p = 0.0005).

**Table 2:** Univariable and multivariable logistical regression of risk factors associated with pulmonary TB.

			Analysis #1 (using ANC above median value)		Analysis #2 (using neutrophilia)	
Risk factor	Unadjusted RR (95%CI)	P-value	Adjusted RR (95%CI)	P-value	Adjusted RR (95%CI)	P-value
**BMI (kg/m2)**						
>25	1.0	–	1.0	–	1.0	–
18–25	1.67 (0.98–2.85)	**0.0019**	1.44 (0.80–2.61)	0.1788	1.50 (0.82–2.75)	0.1689
<18	4.49 (1.98–10.16)		2.39 (0.93–6.15)		2.40 (0.92–6.22)	
**Haemoglobin (g/dL)**						
>12	1.0	–	1.0	–	1.0	–
8–12	2.96 (1.74–5.05)	**<0.0001**	2.52 (1.42–4.48)	**0.0004**	2.76 (1.55–4.92)	**0.0004**
<8	7.83 (3.27–18.72)		5.32 (2.0–14.16)		4.44 (1.60–12.33)	
**Absolute neutrophil count (×10^9^/L)**						
<2.6	1.0	–	1.0	–		
≥2.6	2.76 (1.67–4.55)	**<0.0001**	2.57 (1.48–4.47)	**0.0006**		
**Neutrophilia (ANC >7.5**×**10^9^/L)**						
No	1.0	–			1.0	–
Yes	10.21 (3.94–26.48)	**<0.0001**			6.81 (2.28–20.38)	**0.0005**
**ALT (IU/L)**						
<20	1.0	–				
≥20	1.33 (0.83–2.13)	0.2341				
**Platelets (platelets/uL)**						
<266	1.0	–	1.0		1.0	
≥266	1.63 (1.01–2.63)	**0.0400**	0.92 (0.54–1.59)	0.7670	0.98 (0.58–1.68)	0.9500
**Endotoxin (EU/mL)**						
<0.7	1.0	–				
≥0.7	0.83 (0.51–1.32)	0.4228				
**CD4 cell counts (cells/uL)**						
CD4≥200	1.0	–	1.0	–	1.0	–
CD4 150–199	1.01 (0.49–2.08)	**0.0178**	0.74 (0.33–1.67)	0.3784	0.72 (0.32–1.62)	0.5405
CD4 100–149	1.49 (0.76–2.93)		1.07 (0.50–2.30)		0.90 (0.42–1.94)	
CD4 50–99	1.83 (0.88–3.78)		1.59 (0.71–3.56)		1.25 (0.56–2.81)	
CD4<50	3.00 (1.53–5.92)		1.63 (0.74–3.60)		1.52 (0.68–3.38)	
**Log viral load (log copies/mL)**						
<4.5	1.0	–	1.0	–	1.0	–
≥4.5	2.57 (1.56–4.23)	**0.0001**	1.62 (0.92–2.86)	0.0889	1.70 (0.97–2.99)	0.0632
**WHO stage**						
1 or 2	1.0	–	1.0	–	1.0	–
3 or 4	1.98 (1.25–3.15)	**0.0041**	1.23 (0.69–2.18)	0.4903	1.02 (0.57–1.86)	0.9356

### Characteristics of Patients Grouped by Sputum Mycobacterial Load

Among those with culture-confirmed TB (n = 89), 25 (28.1%) had a low sputum mycobacterial burden (*Xpert-negative/smear-negative*), 40 (44.9%) had an intermediate sputum mycobacterial burden (*Xpert-positive/smear-negative*) and 24 (27.0%) had a high sputum mycobacterial burden (*Xpert-positive/smear-positive*). The median times to positive culture for patients classified as having low, intermediate and high sputum mycobacterial burden were 21 days (IQR, 17–25), 16 days (IQR, 13–20.5) and 9.5 days (IQR, 8–12), respectively (p = 0.0001).

Patients with higher levels of sputum mycobacterial burden had lower BMI, lower hemoglobin concentrations, lower CD4 cell counts and higher viral loads (**Table 1**). Additionally, as the mycobacterial burden among patients increased, so too did the proportion of those with a positive WHO symptom screen, positive LAM ELISA result and those with a radiological abnormality on chest X-ray.

### Neutrophil Counts in Patients Grouped by Sputum Mycobacterial Load

**Figure 3:**
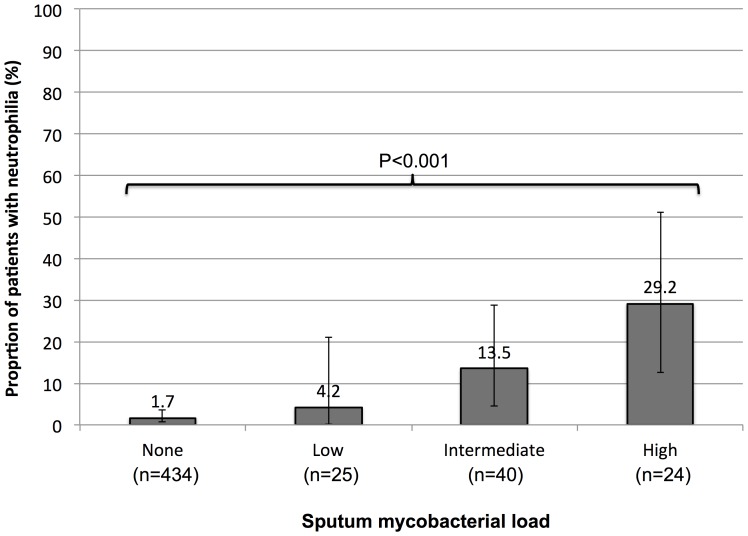
The proportion (with 95% confidence intervals) of patients with HIV-associated TB who have neutrophilia, stratified by increasing level of sputum mycobacterial load.

Blood neutrophil counts were higher in those with greater sputum mycobacterial burden (**Figure 2b**); those who were culture-negative or who had low, intermediate or high mycobacterial burdens had corresponding median ANC values of 2.5×10^9^/L (IQR, 1.8–3.4), 2.9×10^9^/L (IQR, 2.4–6.2), 3.3×10^9^/L (IQR, 2.4–6.2) and 4.4×10^9^/L (IQR, 3.3–7.9), respectively (p = 0.0001). The proportion of patients with neutrophilia also increased with increasing sputum mycobacterial load (**Figure 3**), and nearly 30% of patients with high sputum mycobacterial burden had neutrophilia (p<0.001).

**Figure 4:**
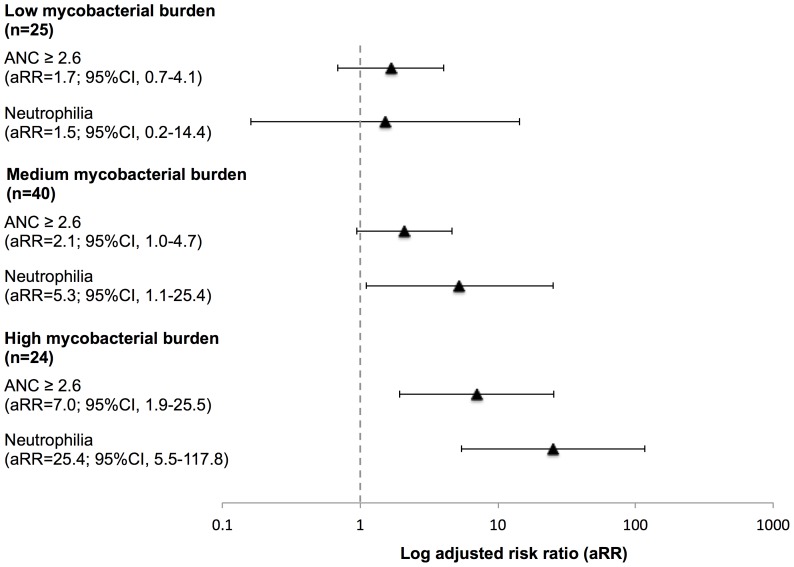
Log adjusted risk ratios (aRR) for increased blood neutrophil counts stratified by low (n = 25), intermediate (n = 40) and high (n = 24) sputum mycobacterial load.

In univariable analyses, sputum mycobacterial load was associated with a large number of variables (using a cutoff of p = 0.1), including an ANC greater than the median value and neutrophilia (**Table S1a)**. In multivariable analyses, the relationship between increasing mycobacterial load and an ANC above median value and neutrophilia persisted (**Tables S1b and S1c**). The fully adjusted risk ratios for an ANC greater than the median value and neutrophilia are displayed in Figure 4 and reveal a strongly positive relationship with increasing mycobacterial load.

## Discussion

Some evidence suggests that neutrophils play a role in both early TB infection and late TB disease although the specific roles in host responses and TB pathology remain incompletely defined. In this study, we found that pulmonary TB disease was independently associated with increased blood neutrophil counts (both an ANC greater than the median value and neutrophilia) and that these parameters were positively associated with sputum mycobacterial load.

The strength of the positive association observed between sputum mycobacterial burden and blood neutrophil counts was particularly striking. This relationship persisted after controlling for important confounding factors including CD4 cell counts. While CD4 cell count was associated with both pulmonary TB and sputum mycobacterial burden in the univariable analyses, these associations did not persist in multivariable analyses. Additionally, we accounted for translocation of gram-negative bacteria and bacterial products from the gastrointestinal tract by measuring endotoxin levels in serum samples. While sputum mycobacterial load levels were independently associated with blood endotoxin levels, there was no clear positive or negative correlation observed between these variables. Since all patients received trimethoprim-sulphamethoxazole prophylaxis, the risk of sepsis as a confounder of increased blood neutrophil counts is further reduced. Having examined for confounding associated with these key variables increases the robustness of our findings.

The results of sputum TB assays were used to categorise patients into four mutually exclusive groups, differing in sputum mycobacterial load. MGIT liquid culture, Xpert MTB/RIF and AFB microscopy all have well described lower limits of detection for MTB [18] and so their results were used in combination to stratify patients. Use of these categories was supported by the finding that higher mycobacterial load groups had shorter times to sputum culture positivity and increased likelihood of positive urine LAM results (two indirect markers that are likely to reflect mycobacterial load). Increasing sputum mycobacterial burden is indicative of poorly controlled pulmonary mycobacterial replication and this was found to be associated with increased neutrophil counts in peripheral blood.

We do not know whether increased neutrophil counts resulted from an increase in absolute numbers of neutrophils in the body or from trafficking of neutrophils into the blood compartment. Increased neutrophil counts are common among patients with active-TB disease in the context of a poorly functioning acquired immune response [1]. While granuloma formation typically involves the interaction of macrophages and lymphocytes, neutrophils are also observed at the site of active mycobacterial disease [2], [19], [20]. Having high peripheral neutrophil counts may be prognostic, where increased blood levels are not only associated with slow clearance of TB bacilli from sputum [5] but also an increased risk of death [6], [7].

Increased blood neutrophil counts in patients with HIV-associated pulmonary TB may be attributable to a number of possible mechanisms [1]. The first includes an immunological loss of control of inflammation, thus allowing for the uncontrolled influx of neutrophils to the site of disease [21] with mobilisation from bone marrow secondary to high circulating levels of inflammatory mediators. For example, patients with active TB have higher circulating levels of IL-8 [22], which drives neutrophil mobilization [23], IL-6, which drives neutrophil granulopoiesis [24], and G-CSF, which performs both of these functions. Systemic activation of the complement cascade, and probably the presence of mycobacterial products in the circulation, would also directly lead to release of neutrophils from marrow stores. Furthermore, during established TB disease in mice, interferon-gamma (IFN-γ) −/− memory CD4+ cells retain antimicrobial activity, but are unable to suppress inflammation and IL-17 production [21]. Because IL-17 can regulate neutrophil mobilisation during MTB infection, high blood neutrophil counts may be observed in patients with advanced TB disease.

Inherent dysfunction of neutrophils in patients with TB may also result in increased blood neutrophil counts. Dysfunctional neutrophils may result in a decreased ability to kill mycobacteria, leading to a non-specific inflammatory cascade, cytokine production or excess necrotic cell death; any or all of which could continue to drive neutrophil accumulation via consequent systemic inflammation [1]. While data are conflicting, in patients with pulmonary TB with and without concomitant HIV-infection, neutrophils may have impaired ability to phagocytose TB bacilli and to undergo oxidative burst [25]–[27].

Finally, neutrophilic ‘compensation’ for an inadequate mononuclear and acquired immune response secondary to advanced HIV disease may also cause increased neutrophil counts peripherally [1]. However, it is not known whether some or all of the mechanisms outlined above contributed to our observation of an ANC greater than the median value and neutrophilia in those with HIV-associated pulmonary TB, as this is beyond the scope of this study. It is also unclear whether the role of neutrophils in host-specific mycobacterial responses is greatly altered in the context of HIV-disease as few studies have investigated this question.

Strengths of this study include a well-characterized, consecutively recruited cohort of patients similar to others in Southern Africa enrolling in ART services, where all pulmonary TB diagnoses were made using a culture-based reference standard. While we have attempted to account for various potential confounding variables including sepsis, residual confounding may still be present. Additionally, while several correlates of mycobacterial burden were assessed, patient sputum MTB burdens were not directly quantified using colony counts. Finally, as this is an observational study, only associations can be reported and immunological mechanisms cannot be explored.

In conclusion, increased blood neutrophil counts were observed in patients with HIV-associated pulmonary TB. Sputum mycobacterial burden was independently associated with an ANC greater than the median value and neutrophilia, where those with a high sputum mycobacterial burden were at greatest risk for having increased blood neutrophil counts. This observation supports the growing body of literature regarding the potential role for neutrophils in the host response to TB and HIV-associated TB. Further studies will be needed to examine these findings in different clinical settings. A greater understanding of the role of neutrophils in the context of TB disease with and without concomitant HIV-disease is needed.

## Supporting Information

Table S1 Logistic regression analyses showing the association between patient characteristics and unadjusted and adjusted risk ratios of varying levels of sputum mycobacterial burden among those with pulmonary TB, where **(a)** is a univariable, multinomial logistic regression with unadjusted risk ratios **(b)** is a multivariable, multinomial logistic regression with adjusted risk ratios (aRR) where the analysis includes an ANC greater than the median value (ANC ≥2.6×10^9^/L) as a potential risk factor and **(c)** is a multivariable, multinomial logistic with aRR where the analysis includes neutrophilia (ANC >7.5×10^9^/L) as a potential risk factor.
(DOCX)
