# Supplementary material for: Blood Neutrophil Counts in HIV-Infected Patients with Pulmonary Tuberculosis: Association with Sputum Mycobacterial Load
Source: PLoS One. 2013 Jul 9;8(7):e67956. doi: 10.1371/journal.pone.0067956 (PMC3706476; doi:10.1371/journal.pone.0067956)
Supplement: Table S1 — Logistic regression analyses showing the association between patient characteristics and unadjusted and adjusted risk ratios of varying levels of sputum mycobacterial burden among those with pulmonary TB, where (a) is a univariable, multinomial logistic regression with unadjusted risk ratios (b) is a multivariable, multinomial logistic regression with adjusted risk ratios (aRR) where the analysis includes an ANC greater than the median value (ANC ≥2.6×10^9^/L) as a potential risk factor and (c) is a multivariable, multinomial logistic with aRR where the analysis includes neutrophilia (ANC >7.5×10^9^/L) as a potential risk factor. (DOCX) [file pone.0067956.s001.docx]

**Table S1a**. Univariable, multinomial logistic regression using logistic regression showing the association between patient characteristics and the unadjusted risk ratio of varying levels of sputum mycobacterial burden among those with pulmonary TB.

|  | **Culture negative (no sputum mycobacterial burden) (n=434)** | **Low sputum mycobacterial burden Unadjusted RR (95%CI) (n=25)** | **Medium sputum mycobacterial burden Undjusted RR (95%CI) (n=40)** | **High sputum mycobacterial burden Unadjusted RR (95%CI) (n=24)** | **P-value** |
| --- | --- | --- | --- | --- | --- |
| **BMI** |  |  |  |  |  |
| >25 | 1.0 | 1.0 | 1.0 | 1.0 | - |
| 18-25 | 1.0 | 1.13 (0.49-2.66) | 3.07 (1.24-7.59) | 1.17 (0.45-3.03) | **0.0029** |
| <18 | 1.0 | 0.84 (0.10-6.98) | 8.86 (2.73-28.75) | 5.42 (1.58-18.56) |  |
| **Haemoglobin (g/dL)** |  |  |  |  |  |
| >12 | 1.0 | 1.0 | 1.0 | 1.0 | - |
| 8-12 | 1.0 | 1.36 (0.58-3.22) | 3.33 (1.50-7.43) | 7.73 (2.23-26.80) | **<0.0001** |
| <8 | 1.0 | 2.73 (0.55-13.44) | 10.00 (3.14-31.83) | 20.00 (4.10-97.64) |  |
| **Absolute netrophil count (x10^9^/L)** |  |  |  |  |  |
| <2.6 | 1.0 | 1.0 | 1.0 | 1.0 | - |
| ≥2.6 | 1.0 | 2.02 (0.87-4.73) | 1.99 (1.00-3.99) | 8.50 (2.50-28.95) | **<0.0001** |
| **Neutrophilia (ANC >7.5 x10^9^/L)** |  |  |  |  |  |
| No | 1.0 | 1.0 | 1.0 | 1.0 |  |
| Yes | 1.0 | 2.46 (0.29-20.84) | 8.84 (2.65-29.43) | 23.29 (7.34-71.91) | **<0.0001** |
| **Platelets (platelets/uL)** |  |  |  |  |  |
| <266 | 1.0 | 1.0 | 1.0 | 1.0 | - |
| ≥266 | 1.0 | 1.81 (0.78-4.24) | 1.28 (0.65-2.51) | 2.18 (0.91-5.20) | 0.1583 |
| **Endotoxin** |  |  |  |  |  |
| <0.7 | 1.0 | 1.0 | 1.0 | 1.0 |  |
| ≥0.7 | 1.0 | 0.44 (0.19-1.02) | 1.52 (0.74-3.11) | 0.61 (0.26-1.44) | **0.0819** |
| **CD4 cell counts (cells/uL)** |  |  |  |  |  |
| CD4 ≥200 | 1.0 | 1.0 | 1.0 | 1.0 | - |
| CD4 150-199 | 1.0 | 1.19 (0.44-3.17) | 1.07 (0.30-3.74) | 0.62 (0.12-3.14) | **0.0114** |
| CD4 100-149 | 1.0 | 0.38 (0.08-1.76) | 2.70 (0.97-7.52) | 2.10 (0.66-6.72) |  |
| CD4 50-99 | 1.0 | 0.57 (0.12-2.65) | 3.58 (1.24-10.34) | 2.09 (0.57-7.68) |  |
| CD4 <50 | 1.0 | 0.98 (0.26-3.68) | 5.67 (2.08-15.45) | 3.61 (1.11-11.72) |  |
| **Baseline viral load, (log copies/mL)** |  |  |  |  |  |
| <4.5 | 1.0 | 1.0 | 1.0 | 1.0 | - |
| ≥4.5 | 1.0 | 0.80 (0.36-1.80) | 4.66 (2.01-10.78) | 5.09 (1.71-15.15) | **<0.0001** |
| **WHO stage at enrolment** |  |  |  |  |  |
| 1 or 2 | 1.0 | 1.0 | 1.0 | 1.0 |  |
| 3 or 4 | 1.0 | 2.05 (0.91-4.60) | 1.81 (0.94-3.49) | 2.22 (0.97-5.06) | **0.0387** |

**Table S1b**. Multivariable, multinomial logistic regression using logistic regression showing the association between patient characteristics and the adjusted risk ratio (aRR) of varying levels of sputum mycobacterial burden among those with pulmonary TB (analysis includes an ANC greater than the median value as a potential risk factor).

|  | **Culture negative (no sputum mycobacterial burden) (n=434)** | **Low sputum mycobacterial burden Adjusted RR (95%CI) (n=25)** | **Medium sputum mycobacterial burden Adjusted RR (95%CI) (n=40)** | **High sputum mycobacterial burden Adjusted RR (95%CI) (n=24)** | **P-value** |
| --- | --- | --- | --- | --- | --- |
| **BMI** |  |  |  |  |  |
| >25 | 1.0 | 1.0 | 1.0 | 1.0 | - |
| 18-25 | 1.0 | 0.82 (0.32-2.13) | 2.80 (1.01-7.74) | 0.90 (0.29-2.75) | 0.1168 |
| <18 | 1.0 | 0.70 (0.07-6.53) | 5.20 (1.25-21.52) | 3.98 (0.86-18.44) |  |
| **Haemoglobin (g/dL)** |  |  |  |  |  |
| >12 | 1.0 | 1.0 | 1.0 | 1.0 | - |
| 8-12 | 1.0 | 1.49 (0.59-3.75) | 3.37 (1.35-8.43) | 4.70 (1.27-17.37) | **0.0020** |
| <8 | 1.0 | 2.29 (0.40-12.99) | 8.36 (1.93-36.31) | 15.33 (2.60-90.44) |  |
| **Absolute neutrophil count (x10^9^/L)** |  |  |  |  |  |
| <2.6 | 1.0 | 1.0 | 1.0 | 1.0 | - |
| ≥2.6 | 1.0 | 1.67 (0.68-4.06) | 2.10 (0.95-4.68) | 7.02 (1.94-25.46) | **0.0019** |
| **Endotoxin (EU/mL)** |  |  |  |  |  |
| <0.7 | 1.0 | 1.0 | 1.0 | 1.0 | - |
| ≥0.7 | 1.0 | 0.40 (0.16-1.00) | 2.04 (0.90-4.63) | 0.65 (0.25-1.72) | **0.0339** |
| **CD4 cell counts (cells/uL)** |  |  |  |  |  |
| CD4 ≥200 | 1.0 | 1.0 | 1.0 | 1.0 | - |
| CD4 150-199 | 1.0 | 1.10 (0.36-3.27) | 0.39 (0.08-2.03) | 0.19 (0.02-1.73) | 0.1265 |
| CD4 100-149 | 1.0 | 0.41 (0.08-1.97) | 1.70 (0.52-5.58) | 1.32 (0.34-5.13) |  |
| CD4 50-99 | 1.0 | 0.67 (0.13-3.38) | 2.66 (0.84-8.45) | 1.43 (0.34-6.07) |  |
| CD4 <50 | 1.0 | 0.48 (0.09-2.46) | 3.08 (0.95-9.92) | 1.29 (0.30-5.59) |  |
| **Baseline viral load, (log copies/mL)** |  |  |  |  |  |
| <4.5 | 1.0 | 1.0 | 1.0 | 1.0 | - |
| ≥4.5 | 1.0 | 0.63 (0.25-1.63) | 2.83 (1.11-7.17) | 2.98 (0.90-9.93) | **0.0246** |
| **WHO stage at enrolment** |  |  |  |  |  |
| 1 or 2 | 1.0 | 1.0 | 1.0 | 1.0 | - |
| 3 or 4 | 1.0 | 2.31 (0.90-5.94) | 0.78 (0.32-1.89) | 1.00 (0.34-2.98) | 0.3396 |

**Table S1c.** Multivariable, multinomial logistic regression using logistic regression showing the association between patient characteristics and the adjusted risk ratio (aRR) of varying levels of sputum mycobacterial burden among those with pulmonary TB (analysis includes neutrophilia as a potential risk factor).

|  | **Culture negative (no sputum mycobacterial burden) (n=434)** | **Low sputum mycobacterial burden Adjusted RR (95%CI) (n=25)** | **Medium sputum mycobacterial burden Adjusted RR (95%CI) (n=40)** | **High sputum mycobacterial burden Adjusted RR (95%CI) (n=24)** | **P-value** |
| --- | --- | --- | --- | --- | --- |
| **BMI** |  |  |  |  |  |
| >25 | 1.0 | 1.0 | 1.0 | 1.0 | - |
| 18-25 | 1.0 | 0.82 (0.32-2.13) | 2.76 (0.99-7.71) | 0.91 (0.28-2.96) | 0.1295 |
| <18 | 1.0 | 0.70 (0.08-6.58) | 4.94 (1.19-20.61) | 4.22 (0.86-20.57) |  |
| **Haemoglobin (g/dL)** |  |  |  |  |  |
| >12 | 1.0 | 1.0 | 1.0 | 1.0 | - |
| 8-12 | 1.0 | 1.56 (0.62-3.94) | 3.59 (1.43-9.00) | 6.48 (1.65-25.43) | **0.0024** |
| <8 | 1.0 | 2.27 (0.40-12.73) | 7.34 (1.63-33.05) | 10.37 (1.46-73.43) |  |
| **Neutrophilia (ANC >7.5 x10^9^/L)** |  |  |  |  |  |
| No | 1.0 | 1.0 | 1.0 | 1.0 | - |
| Yes | 1.0 | 1.51 (0.16-14.36) | 5.27 (1.10-25.34) | 25.36 (5.46-117.77) | **0.0004** |
| **Endotoxin (EU/mL)** |  |  |  |  |  |
| <0.7 | 1.0 | 1.0 | 1.0 | 1.0 | - |
| ≥0.7 | 1.0 | 0.41 (0.17-1.02) | 2.05 (0.91-4.66) | 0.59 (0.21-1.65) | **0.0303** |
| **CD4 cell counts (cells/uL)** |  |  |  |  |  |
| CD4 ≥200 | 1.0 | 1.0 | 1.0 | 1.0 | - |
| CD4 150-199 | 1.0 | 1.09 (0.36-3.25) | 0.37 (0.07-1.93) | 0.14 (0.01-1.40) | 0.1601 |
| CD4 100-149 | 1.0 | 0.37 (0.08-1.78) | 1.49 (0.46-4.86) | 0.80 (0.19-3.27) |  |
| CD4 50-99 | 1.0 | 0.60 (0.12-3.00) | 2.17 (0.69-6.86) | 0.85 (0.20-3.60) |  |
| CD4 <50 | 1.0 | 0.45 (0.09-2.28) | 2.87 (0.89-9.26) | 1.09 (0.24-4.94) |  |
| **Baseline viral load, (log copies/mL)** |  |  |  |  |  |
| <4.5 | 1.0 | 1.0 | 1.0 | 1.0 | - |
| ≥4.5 | 1.0 | 0.66 (0.26-1.68) | 2.87 (1.14-7.29) | 3.35 (0.96-11.69) | **0.0197** |
| **WHO stage at enrolment** |  |  |  |  |  |
| 1 or 2 | 1.0 | 1.0 | 1.0 | 1.0 | - |
| 3 or 4 | 1.0 | 2.35 (0.90-6.14) | 0.69 (0.28-1.72) | 0.58 (0.17-2.03) | 0.2183 |
